# Outdoor Particulate Matter Exposure and Lung Cancer: A Systematic Review and Meta-Analysis

**DOI:** 10.1289/ehp/1408092

**Published:** 2014-06-06

**Authors:** Ghassan B. Hamra, Neela Guha, Aaron Cohen, Francine Laden, Ole Raaschou-Nielsen, Jonathan M. Samet, Paolo Vineis, Francesco Forastiere, Paulo Saldiva, Takashi Yorifuji, Dana Loomis

**Affiliations:** 1 International Agency for Research on Cancer, Lyon, France; 2 Health Effects Institute, Boston, Massachusetts, USA; 3 Channing Division of Network Medicine, Department of Medicine, Brigham and Women’s Hospital and Harvard Medical School, Boston, Massachusetts, USA; 4 Department of Environmental Health, and; 5 Department of Epidemiology, Harvard School of Public Health, Boston, Massachusetts, USA; 6 Institute of Cancer Epidemiology, Danish Cancer Society, Copenhagen, Denmark; 7 Department of Preventive Medicine, Keck School of Medicine, University of Southern California, Los Angeles, California, USA; 8 Department of Epidemiology and Public Health, Imperial College London, London, United Kingdom; 9 Department of Epidemiology, Regional Health Authority, Lazio, Roma, Italy; 10 Department of Pathology, School of Medicine, University of São Paulo, São Paulo, Brazil; 11 Department of Human Ecology, Graduate School of Environmental and Life Sciences, Okayama University, Okayama, Japan

## Abstract

Background: Particulate matter (PM) in outdoor air pollution was recently designated a Group I carcinogen by the International Agency for Research on Cancer (IARC). This determination was based on the evidence regarding the relationship of PM_2.5_ and PM_10_ to lung cancer risk; however, the IARC evaluation did not include a quantitative summary of the evidence.

Objective: Our goal was to provide a systematic review and quantitative summary of the evidence regarding the relationship between PM and lung cancer.

Methods: We conducted meta-analyses of studies examining the relationship of exposure to PM_2.5_ and PM_10_ with lung cancer incidence and mortality. In total, 18 studies met our inclusion criteria and provided the information necessary to estimate the change in lung cancer risk per 10-μg/m^3^ increase in exposure to PM. We used random-effects analyses to allow between-study variability to contribute to meta-estimates.

Results: The meta-relative risk for lung cancer associated with PM_2.5_ was 1.09 (95% CI: 1.04, 1.14). The meta-relative risk of lung cancer associated with PM_10_ was similar, but less precise: 1.08 (95% CI: 1.00, 1.17). Estimates were robust to restriction to studies that considered potential confounders, as well as subanalyses by exposure assessment method. Analyses by smoking status showed that lung cancer risk associated with PM_2.5_ was greatest for former smokers [1.44 (95% CI: 1.04, 2.01)], followed by never-smokers [1.18 (95% CI: 1.00, 1.39)], and then current smokers [1.06 (95% CI: 0.97, 1.15)]. In addition, meta-estimates for adenocarcinoma associated with PM_2.5_ and PM_10_ were 1.40 (95% CI: 1.07, 1.83) and 1.29 (95% CI: 1.02, 1.63), respectively.

Conclusion: The results of these analyses, and the decision of the IARC Working Group to classify PM and outdoor air pollution as carcinogenic (Group 1), further justify efforts to reduce exposures to air pollutants that can arise from many sources.

Citation: Hamra GB, Guha N, Cohen A, Laden F, Raaschou-Nielsen O, Samet JM, Vineis P, Forastiere F, Saldiva P, Yorifuji T, Loomis D. 2014. Outdoor particulate matter exposure and lung cancer: a systematic review and meta-analysis. Environ Health Perspect 122:906–911; http://dx.doi.org/10.1289/ehp.1408092

## Introduction

Outdoor air pollution is a complex mixture containing a number of known carcinogens and has been associated with increased lung cancer risk in many studies over the past 50 years. Past reviews of the body of evidence regarding outdoor and household air pollution indicated that both were associated with lung cancer risk; specifically, exposures to increased levels of particles, as well as other indices of air pollution, were associated with increased lung cancer risk. However, the evidence was considered inconclusive regarding which specific components of the air pollution mixture are driving the increased risk (Samet and Cohen 2006). The International Agency for Research on Cancer (IARC) recently concluded that exposure to outdoor air pollution and to particulate matter (PM) in outdoor air is carcinogenic to humans (IARC Group 1) and causes lung cancer (IARC, in press; Loomis et al. 2013). Epidemiological studies of long-term residential exposure to outdoor air pollution in terms of PM played a critical role in IARC’s evaluation.

In this manuscript, which originated with the IARC review, we provide meta-analyses of the lung cancer risk associated with exposure to PM in outdoor air, specifically PM_2.5_ (particles with aerodynamic diameter ≤ 2.5 μm, or fine particles) and PM_10_ (≤ 10 μm, or inhalable particles). We performed analyses in subgroups defined by geographic region, potential confounders and effect modifiers, and exposure assessment method. We also examined the influence of single studies to the overall meta-estimate.

## Methods

*Literature search*. The studies included in this analysis were a key component of the epidemiological evidence reviewed by the IARC Working Group in its evaluation of the carcinogenicity of PM, as reported in *IARC Monograph* 109 (IARC, in press; Loomis et al. 2013). Relevant studies were identified in several stages, beginning with a systematic search of PubMed (http://www.ncbi.nlm.nih.gov/pubmed/) using the keywords “air pollution OR particulate matter OR traffic AND cancer” in the title or abstract, with the results restricted to studies of humans. An initial search was conducted in December 2012 and updated automatically through October 2013. This search retrieved 604 studies.

Abstracts of the papers retrieved in the electronic search were screened manually for relevance to the topic of the *IARC Monograph* on outdoor air pollution. Ecological studies, with data on both outcome and exposure collected at the aggregate level, were excluded because of the inherent limitations of such studies. Instead, we considered all cohort and case–control studies available that provided individual outcome information and—in many cases—individual measures of exposure. The reference lists of the papers judged to be relevant at this stage were then searched for other potentially relevant papers, which were screened in turn. Members of the working group who were familiar with the research identified three additional studies that were in press at the time of the electronic search. Through this process, 201 potentially relevant papers were identified. Electronic full-text copies of those papers were made available to members of the working group, who reviewed the search results and the papers in detail and selected those studies considered relevant for inclusion in the *IARC Monograph*.

*Inclusion and exclusion criteria*. Studies were included in the current meta-analysis if they provided quantitative estimates of residential exposure to PM_2.5_ and/or PM_10_. Further, studies were required to provide quantitative estimates of the change in lung cancer incidence or mortality associated with exposure to either indicator of PM; this could be reported as the change in risk per microgram per cubic meter or per quantile of exposure. Studies that reported results for the association of lung cancer with other air pollutants or exposure to traffic but did not provide quantitative estimates for PM were not included in the meta-analysis.

We considered lung cancer mortality and incidence studies together because mortality is a valid indicator of incidence. Survival rates provided by the Surveillance, Epidemiology, and End Results (SEER) program from 2003 to 2009 estimate 5-year survival rates among U.S. white males and females at 14.5% and 19.5%, respectively (Howlader et al. 2013). Because the case–fatality rate is high for lung cancer, mortality and incidence are comparable; thus, it is reasonable to include both outcomes within the same meta-analysis.

Where multiple publications included overlapping study populations, we included the publication that considered the largest number of cases and/or that evaluated results based on the longest follow-up. In addition, we did not place any restrictions based on whether or not a study adjusted for specific confounders. All studies were adjusted for the effect of age and sex; however, the sets of other potential confounders for which adjustments were made varied by study. Thus, the sensitivity of estimates to confounder adjustment was considered. All risk estimates were abstracted by one of the authors, reviewed by the IARC Working Group, and double-checked for accuracy by a second author.

*Statistical analyses*. All study estimates were converted to represent the change in lung cancer risk per 10-μg/m^3^ unit increase in exposure to PM_2.5_ or PM_10_. If we could not reliably convert the values in a particular study to the aforementioned units, we contacted the authors of the original study for further information. If information necessary to convert estimates could not be obtained, the study was excluded from consideration.

Estimates from the studies were combined using a random-effects model, which allowed between-study heterogeneity to contribute to the variance (DerSimonian and Laird 1986). *I*^2^ values are reported, representing the estimated percent of the total variance that is explained by between-study heterogeneity (Higgins et al. 2003). We also conducted chi-square tests of homogeneity to compare meta-estimates divided into subgroups by region, smoking status, and exposure assessment method. In some cases, individual studies reported results only for subgroups, such as by sex or time period of study. Such stratified estimates were first combined into a single, study-specific estimate using fixed-effects regression and then included in analyses to obtain the overall meta-estimate. In addition, studies restricted to certain subgroups were considered to have been adjusted for possible confounding by the subgroup of interest; for example, studies among men only were considered to have accounted for the confounding effects of sex. Finally, forest and funnel plots were created to provide a visual summary of the distribution of study-specific effect estimates. In lieu of statistical tests of funnel plot asymmetry, we conducted trim and fill analyses. The trim and fill analysis method removes the smallest studies until a symmetric funnel plot is obtained; then, those removed studies are added back with their hypothetical “counterparts” to recalculate the meta-estimate that would have been obtained from a symmetric funnel plot (Duval and Tweedie 2000; Higgins et al. 2008). Analyses were conducted using STATA (v12.1; StataCorp, College Station, TX, USA).

## Results

*Studies included*. We identified 17 cohort studies (Beelen et al. 2008; Beeson et al. 1998; Cao et al. 2011; Carey et al. 2013; Cesaroni et al. 2013; Hales et al. 2012; Hart et al. 2011; Heinrich et al. 2013; Jerrett et al. 2013; Katanoda et al. 2011; Krewski et al. 2009; Lepeule et al. 2012; Lipsett et al. 2011; McDonnell et al. 2000; Naess et al. 2007; Pope et al. 2002; Raaschou-Nielsen et al. 2013) and one case–control study (Hystad et al. 2013) of lung cancer that provided estimates of the quantitative relationships between the risk of lung cancer and exposure to PM_2.5_ or PM_10_ that could be expressed per 10-μg/m^3^ change in PM. Estimates from one cohort study (Naess et al. 2007) could not be converted to units of 10-μg/m^3^, and thus, this study did not contribute to the meta-estimates. In addition, a recently accepted paper (Puett et al. 2014) was included in this analysis because it met the criteria for inclusion.

Table 1 summarizes the 18 studies included in these analyses. In total, there were 14 and 9 studies that provided estimates of the lung cancer risk associated with exposure to PM_2.5_ and to PM_10_, respectively. There were four studies from Europe, eight studies from North America, and two studies from other regions that contributed to the overall meta-estimates for PM_2.5_. Regarding PM_10_, there were three European studies, five North American studies, and one study from another region that contributed to the overall meta-estimates.

**Table 1:** Summary of studies included in meta-analyses of lung cancer risk associated with exposure to particulate matter.

Continent	Study ID	Reference	No. of events	Total population	Study period	Exposure assessment method	Exposure distribution (mean ± SD)	Study
North America
California, USA	1	Beeson et al. 1998	16 (incidence)	6,338	1977–1992	Fixed site monitor	PM_10_: 51.0 ± 16.5	AHSMOG
California, USA	2	McDonnell et al. 2000	13 (mortality)	3,769	1977–1992	Fixed site monitor	PM_2.5_: 31.9 ± 10.7	AHSMOG
United States	3	Pope et al. 2002	NA	415,000	1982–1998	Fixed site monitor	PM_10_: 28.8 ± 5.9	ACS-CPS II
United States	4	Krewski et al. 2009	9,788 (mortality)	499,968	1982–2000	Fixed site monitor	PM_2.5_: 21.2 ± 10.8 (1979–1983) PM_2.5_: 14.0 ± 9.1 (1999–2000)	ACS-CPS II
Los Angeles, CA, United States	5	Jerrett et al. 2013	1,481 (mortality)	73,711	1982–2000	Land use regression	PM_2.5_: 14.1 ± 12.4	ACS-CPS II
United States	6	Hart et al. 2011	800 (mortality)	53,814	1985–2000	Inverse distance weighting (PM_2.5_)/spatio­temporal (PM_10_)	PM_2.5_: 14.1 ± 4.0 PM_10_: 26.8 ± 6.0	TrIPS
California, USA	7	Lipsett et al. 2011	275 (PM_10_), 234 (PM_2.5_) (mortality)	101,784 (PM_10_), 73,489 (PM_2.5_)	1997–2005	Inverse distance weighting	PM_2.5_: 15.6 ± 4.5 PM_10_: 29.2 ± 9.8	CTS
United States	8	Lepeule et al. 2012	632 (mortality)	8,096	1975–2009	Fixed site monitor	PM_2.5_: 15.9^*b*^	Harvard Six Cities Study
Canada	9	Hystad et al. 2013	2,390 (incidence)	5,897	1994–1997	Spatio­temporal model	PM_2.5_: 11.9 ± 3.0	National Enhanced Cancer Surveillance System Case-Control study
United States	10	Puett et al. 2014	1,648 (incidence)	97,865	1998–2010	Spatio­temporal model	PM_2.5_: 13.1 ± 3.0 PM_10_: 21.6 ± 6.0	NHS
Europe
Netherlands	11	Beelen et al. 2008	1,940 (incidence)	120,852	1986–1997	Land use regression	PM_2.5_: 28.3 ± 2.1	Netherlands Cohort study of Diet and Cancer.
United Kingdom	12	Carey et al. 2013	5,273 (mortality)	830,842	2003–2007	Air dispersion	PM_2.5_: 12.9 ± 1.4 PM_10_: 19.7 ± 2.3	Clinical Practice Research Datalink
Italy	13	Cesaroni et al. 2013	12,208 (mortality)	1,265,058	2001–2010	Air dispersion	PM_2.5_: 23.0 ± 4.4	Rome Longitudinal Study
Germany	14	Heinreich et al. 2013	41 (mortality)	4,752	1980–2008	Fixed site monitor	PM_10_: 43.7^*b*^	German Women’s Health Study
European Union	15	Raaschou-Neilsen et al. 2013	2,095 (incidence)	312,944	1990s	Land use regression	PM_2.5_: 13.4 ± 1.2 PM_10_: 21.3 ± 2.7	ESCAPE
Other
China	16	Cao et al. 2011	624 (mortality)	70,947	1991–2000	Fixed site monitor	PM_2.5_:^*a*^	China National Hypertension follow-up survey
Japan	17	Katanoda et al. 2011	421 (mortality)	63,520	1983–1995	Fixed site monitor	PM_2.5_: 28.8^*b*^	Three Prefecture Cohort Study
New Zealand	18	Hales et al. 2012	1,686 (mortality)	1,050,222	1996–1999	Land use regression	PM_10_: 8.3 ± 8.4	New Zealand Census Mortality Study
Abbreviations: AHSMOG, Adventist Health Study on Smog; ACS-CPS II, American Cancer Society Cancer Prevention Study II; CTS, California Teachers Study; ESCAPE, European Study of Cohorts for Air Pollution Effects; NA, not available; NHS, Nurses’ Health Study; TrIPS, Trucking Industry Particle Study. ^***a***^Mean and SD of PM_2.5_ for Cao et al. (2011) could not be obtained. The numbers reported represent the range of exposure estimated by converting TSP to PM_2.5_ with a 3:1 ratio. ^***b***^SD not reported.								

Jerrett et al. (2013) studied a subset of the full American Cancer Society Cancer Prevention Study II (ACS-CPS II) cohort considered by Krewski et al. (2009). However, Jerrett et al. (2013) used land use regression to estimate PM, whereas Krewski et al. (2009) used fixed site monitors; furthermore, each study considered different confounders in their final analyses. Therefore, we excluded Jerrett et al. (2013) from all analyses where it would overlap with Krewski et al. (2009).

**Figure 1:**
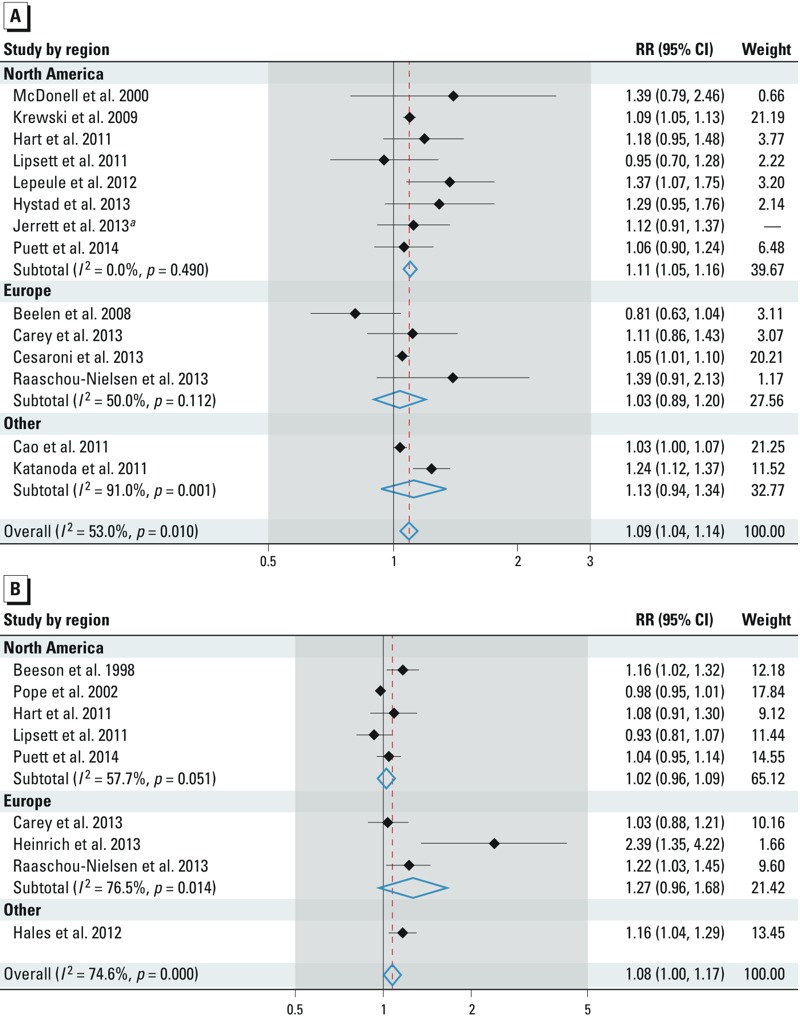
Estimates of lung cancer risk associated a 10-μg/m^3^ change in exposure to PM_2.5_ (*A*) and PM_10_ (*B*) overall and by geographic region of study. Weights represent the contribution of each study effect estimate to the overall meta-estimate. ***^a^***Jerrett et al. (2013) contributes neither to the overall nor to the continent-specific meta-estimates; it is only included here for visualization.

*Overall meta-estimates for PM_2.5_ and PM_10_*_._ Figure 1 presents the estimated effect for each study, grouped by the continent where the study was conducted; Jerrett et al. (2013) is included in Figure 1 for visualization, but it does not contribute to overall or continent-specific meta-estimates. All estimates represent the change in the risk of lung cancer mortality/incidence associated with a 10-μg/m^3^ increase in PM_2.5_ or PM_10_. The meta-relative risk [95% confidence interval (CI)] for lung cancer associated with PM_2.5_ was 1.09 (95% CI: 1.04, 1.14). The meta-relative risk of lung cancer associated with PM_10_ was similar, but less precise: 1.08 (95% CI: 1.00, 1.17). When restricting our analysis to cohort studies that examined both measures of PM [the Adventist Health Study on Smog (AHSMOG) study, ACS-CPS II, Trucking Industry Particle Study (TrIPS), California Teachers Study (CTS), Nurses’ Health Study (NHS), Clinical Practice Research Datalink, and European Study of Cohorts for Air Pollution Effects (ESCAPE)], the meta-estimates associated with PM_2.5_ and PM_10_ are 1.09 (95% CI: 1.06, 1.13) and 1.07 (95% CI: 0.98, 1.15), respectively. Random-effects estimation for these values may suggest inconsistencies between studies. The between-study variance for PM_2.5_ and PM_10_ were 56.4% and 74.6% of the total variance, respectively. Chi-square tests of homogeneity provided little evidence of difference between continent-specific meta-estimates for PM_2.5_ (*p* = 0.656) and modest evidence of heterogeneity by continent-specific meta-estimates for PM_10_ (*p* = 0.074).

Funnel plots for both PM_2.5_ and PM_10_ were visually asymmetrical (see Supplemental Material, Figure S1); thus, trim and fill analyses were conducted to test the volume of information that would be necessary to construct a symmetrical funnel plot. With respect to PM_2.5_, the estimate accounting for funnel plot asymmetry was 1.08 (95% CI: 1.03, 1.13); this estimate required trimming and filling in the funnel plot with three hypothetical studies. With regard to PM_10_, the estimate accounting for funnel plot asymmetry was 1.00 (95% CI: 0.92, 1.08); this estimate required filling in the funnel plot with four hypothetical studies. In addition, we conducted influence analyses to determine if any specific study highly influenced the overall meta-estimate. Results showed that, overall, meta-estimates were not reliant on inclusion of any specific study; confidence intervals for meta-estimates excluding one study at a time overlapped, and meta-estimates consistently supported a positive association between PM exposure and lung cancer incidence and mortality (see Supplemental Material, Table S1).

*Subgroup analyses*. Table 2 presents subgroup analyses by continent, exposure assessment method, and smoking status; in addition, meta-estimates excluding studies that did not adjust for confounders of interest are presented. Region-specific meta-estimates are also summarized, with individual study estimates, in Figure 1. The PM_2.5_ meta-estimates for Europe, North America, and other continents were 1.03 (95% CI: 0.89, 1.20), 1.11 (95% CI: 1.05, 1.16), and 1.13 (95% CI: 0.94, 1.34), respectively (Figure 1A). Although these estimates show a slight variation, their confidence intervals are largely overlapping, and homogeneity tests suggest no evidence of differences across regions. Regarding PM_10_, the meta-estimates for Europe and North America are 1.27 (95% CI: 0.96, 1.68) and 1.02 (95% CI: 0.96, 1.09), respectively (Figure 1B). The estimate from North America, based on five studies, was more precise and less suggestive of a relationship between PM_10_ and lung cancer risk. A study in New Zealand (Hales et al. 2012) was the only one available outside of Europe and North America; the reported estimate from this study was 1.16 (95% CI: 1.04, 1.29). Estimates by continent do not appear to be heterogeneous (Table 2).

**Table 2:** Estimates for the relationship between a 10-μg/m^3^ change in PM_2.5_ and PM_10_ exposure and lung cancer risk.

Exposure	RR (95% CI)	*I*^2^ (*p*-value)	Homogeneity test^*a*^	Studies included (by ID)^*b*^
PM_2.5_
Full meta-estimate	1.09 (1.04, 1.14)	56.4% (0.007)		All
Continent
North America	1.11 (1.05, 1.16)	6.5% (0.378)		2, 4, 6, 7, 8, 9, 10
Europe	1.03 (0.89, 1.20)	50.0% (0.112)		11, 12, 13, 15
Others	1.13 (0.94, 1.34)	91.0% (0.001)	*p *= 0.656	16, 17
Exposure assessment method
Fixed site monitor	1.12 (1.04, 1.21)	77.1% (0.002)		2, 4, 8, 16, 17
Other	1.06 (1.00, 1.13)	16.2% (0.298)	*p *= 0.268	5, 6, 7, 9, 10, 11, 12, 13, 15
Smoking status
Never	1.18 (1.00, 1.39)	0.0% (0.928)		3, 7, 8, 9, 10, 15
Former	1.44 (1.04, 2.01)	66.3% (0.031)		3, 8, 9, 15
Current	1.06 (0.97, 1.15)	0.0% (0.544)	*p *= 0.197	3, 8, 9, 15
Confounder adjustment
Smoking status	1.10 (1.04, 1.17)	61.4% (0.004)		2, 4, 7, 8, 9, 10, 11, 12, 15, 16, 17
SES/income	1.04 (0.96, 1.12)	24.2% (0.252)		5, 7, 10, 11, 13, 15
Education	1.07 (1.03, 1.11)	37.7% (0.117)		4, 8, 9, 10, 12, 13, 15, 16,
Occupation	1.08 (1.05, 1.11)	0.4% (0.420)		4, 6, 7, 9, 10, 13, 15
PM_10_
Full meta-estimate	1.08 (1.00, 1.17)	74.6% (> 0.001)		All
Continent
North America	1.02 (0.96, 1.09)	57.7% (0.051)		1, 3, 6, 7, 10
Europe	1.27 (0.96, 1.68)	76.5% (0.014)		12, 14, 15
Others	1.16 (1.04, 1.29)	—	*p *= 0.074	18
Exposure assessment method
Fixed site monitor
Other	1.17 (0.93, 1.47)	87.3% (> 0.000)		1, 3, 14
Smoking status	1.07 (0.99, 1.15)	43.9% (0.113)	*p *= 0.484	6, 7, 10, 12, 15, 18
Never
Former	1.11 (0.94, 1.33)	0.0% (0.407)		7, 10, 15
Current	—	—		—
Confounder adjustment				
Smoking status	1.08 (0.99, 1.17)	77.2% (> 0.001)		1, 3, 7, 10, 12, 14, 15, 18
SES/income	1.08 (0.97, 1.20)	65.5% (0.033)		7, 10, 15, 18
Education	1.11 (1.01, 1.21)	79.7% (> 0.001)		1, 3, 10, 12, 14, 15, 18
Occupation	1.02 (0.95, 1.10)	56.7% (0.055)		3, 6, 7, 10, 15
Estimates are the result of random-effects meta-analysis. RR, meta-relative risk. ^***a***^*p*-Value based on a chi-square distribution. ^***b***^Studies included in the analysis according to ID numbers listed in Table 1. Results from Naess et al. (2007) could not be converted to 10-μg/m^3^ units, and were, thus, excluded. The change in lung cancer mortality associated with a 1-quartile increase in PM_2.5_ and PM_10_ were identical, 1.26 (95% CI: 1.23, 1.28).				

With regard to exposure assessment method, meta-estimates from studies using fixed site monitors were compared to those from studies using modeling-based estimation techniques, for example, land use regression. For both PM_2.5_ and PM_10_ exposure, the meta-estimate from studies using fixed site monitors was higher than the meta-estimate from studies using modeling-based exposure assessment techniques. However, homogeneity tests for PM_2.5_ and PM_10_ (*p* = 0.268 and *p* = 0.484, respectively) suggested no difference between exposure assessment method subgroups (Table 2).

We also conducted analyses by subgroups of current, former, and never-smokers. The meta-estimate for lung cancer risk associated with PM_2.5_ was greatest for former smokers, 1.44 (95% CI: 1.04, 2.01) followed by never-smokers, 1.18 (95% CI: 1.00, 1.39), and then current smokers, 1.06 (95% CI: 0.97, 1.15). A test of homogeneity suggested no evidence of difference between subgroups (*p* = 0.197); a lack of statistical power to detect differences may have contributed to this finding. The meta-estimate for lung cancer risk associated with PM_10_ for never-smokers was 1.11 (95% CI: 0.94, 1.33). Estimates for current and former smokers were only available from one study, Raaschou-Nielsen et al. (2013), and were 1.27 (95% CI: 1.02, 1.58) and 0.98 (95% CI: 0.67, 1.44), respectively.

Table 2 summarizes meta-estimates by subgroups of studies that account for confounding by smoking status, socioeconomic status (SES)/income, education, and occupation (which includes occupational exposure). The magnitude of the meta-estimates of lung cancer risk associated with PM_2.5_ varied modestly but remained elevated with various adjustments. The meta-estimates of lung cancer risk associated with PM_10_ exposure behaved similarly, but there was some indication of greater sensitivity to the control of covariates, particularly occupation.

*Histologic subtypes*. Table 3 provides estimates of the relationship between PM_2.5_ and PM_10_ and the two most frequent histological subtypes of lung cancer: adenocarcinoma and squamous cell carcinoma. The meta-estimates for adenocarcinoma associated with PM_2.5_ and PM_10_ were 1.40 (95% CI: 1.07, 1.83) and 1.29 (95% CI: 1.02, 1.63), respectively. The meta-estimate of the relationship between squamous cell carcinoma and PM_2.5_ is 1.11 (95% CI: 0.72, 1.72). The relationship between PM_10_ and squamous cell carcinoma was examined in only one study (Raaschou-Nielsen et al. 2013) in our review, which reported a meta-relative risk of 0.84 (95% CI: 0.50, 1.41) per 10 μg/m^3^.

**Table 3:** Estimates for the relationship between a 10-μg/m^3^ change in PM_2.5_ and PM_10_ and histological cancer subtypes.

Exposure and outcome	RR (95% CI)	*n*	Studies included (by ID)^*a*^
PM_2.5_
Adenocarcinoma	1.40 (1.07, 1.83)	2,339	9, 10, 15
Squamous cell carcinoma	1.11 (0.72, 1.72)	1,523	9, 15
PM_10_
Adenocarcinoma	1.29 (1.02, 1.63)	965	10, 15
Squamous cell carcinoma	—	—	—
RR, meta-relative risk. Estimates are the result of random-effects meta-analysis. ^***a***^Studies included in the analysis according to ID numbers listed in Table 1.			

## Discussion

We conducted meta-analyses of the relationship between exposure to ambient PM and lung cancer incidence and mortality. Meta-estimates combine incidence and mortality studies due to the high fatality rate among incident lung cancers. These quantitative analyses complement the qualitative classification of the evidence by the Monograph 109 Working Group (IARC, in press). Most of the data were obtained from cohort studies, and our analytical results are similar across diverse study populations, potential confounders considered, as well as exposure assessment methods; this consistency supports the IARC Working Group’s conclusion that PM from outdoor air pollution is a Group 1 carcinogen and causes lung cancer. Air pollution is ubiquitous, and all populations are exposed to it at some level, albeit with considerable variation between the most and the least polluted areas (Brauer et al. 2012). Thus, these results are important for policy makers and public health practitioners across the world.

In this analysis, we focused attention on PM_2.5_ and PM_10_, which are prominent components of the ambient air pollution mixture. Of course, PM_10_ includes the PM_2.5_ size fraction; however, these particle size groups are believed to differ in regard to human health effects. PM_2.5_ includes a higher proportion of mutagenic species (Buschini et al. 2001; Valavanidis et al. 2008), many of which are products of combustion (Brauer et al. 2001). Further, smaller particles penetrate more deeply into the lung and are more likely to be retained (Stuart 1976). On the other hand, the coarse fraction of the PM_10_-size group consists mainly of minerals and biologic materials (Valavanidis et al. 2008). Thus, PM_2.5_ is generally believed to be most relevant to health effects, including cancer.

A number of potential confounders are often considered when examining the relationship between PM and lung cancer risk, the most important overall being tobacco smoking. Meta-estimates for the relationship of PM_2.5_ to lung cancer were consistent with the overall meta-estimate when restricting to studies that considered confounding by smoking status, SES/income, education, occupation, or sex. In addition, analyses by continent of study (Europe, North America, or other) yielded consistent, positive associations between PM_2.5_ and lung cancer. For PM_10_, the data were less abundant and the findings of sensitivity analyses were less robust than for PM_2.5_.

We did not conduct statistical tests for assessment of publication bias, because these tests are specific to randomized controlled trials (Sterne et al. 2011) and rely on assumptions that are not applicable to meta-analyses of observational research (Egger et al. 1998). We conducted trim and fill analyses, which require a strong assumption that a funnel plot should be symmetrical and that there is no between-study variance (Duval and Tweedie 2000; Higgins et al. 2008). Conclusions regarding the relationship between PM_2.5_ and lung cancer risk were robust. Trim and fill analyses for PM_10_ and lung cancer risk led to a null-centered estimate; however, this required trimming and filling four out of 10 studies. In short, a large number of hypothetical studies would be required to construct a symmetrical funnel plot and change the results of our meta-analyses. In addition, results for lung cancer risk from PM_2.5_ and PM_10_ were robust to influence analyses, where the meta-estimate was recalculated with the systematic exclusion of each study.

Exposure assessment techniques differed across studies. Some studies used fixed site monitors, and others employed more complex modeling approaches; regardless of the method used, all individuals in a study are assigned an estimate of individual-level exposure. Modeling techniques, such as land use regression and air dispersion, attempt to provide residential estimates of exposure to PM, whereas fixed site monitor techniques indicate group-level exposures without further modeling. In fact, none of the exposure assessment methods used provides a true, individual-level measure of exposure to PM. Meta-estimates of lung cancer risk associated with both PM_2.5_ and PM_10_ from studies using fixed site monitors were slightly higher than those obtained from studies using advanced exposure modeling methods. However, homogeneity tests suggest no difference in estimates between exposure assessment techniques.

In addition to adjusting for smoking status, some studies provided analyses for PM_2.5_ by smoking subgroups allowing assessment–differential effects of PM by smoking status (current, former, and never-smokers). Data were limited for examining the relationship between PM_10_ and lung cancer risk: Only three studies provided any subgroup-specific information, which pertained only to never-smokers. Six studies provided information on the relationship between PM_2.5_ and lung cancer by current, former, or never-smoking status, and one presented results specific to never-smokers. Meta-estimates from these studies suggest that never- and former smokers may have an elevated risk of lung-cancer associated with PM_2.5_ compared to current smokers; however, even current smokers exhibited a modest, but imprecise, elevated risk of lung cancer due to PM_2.5_. Further, these results are limited by lack of detailed information on patterns of former smoking. We are not able to disentangle effects of air pollution on lung cancer risk between former heavy versus former light smokers, which might be expected to differ. Homogeneity tests did not provide support for different effects by smoking subgroups.

In a recent study of a large cohort of U.S. non-metal miners, Silverman et al. (2012) reported a dose–response curve that was steeper at lower exposures to respirable elemental carbon (a marker for diesel exhaust) and leveled off at higher exposures. In that study, the authors observed reduced diesel exhaust–associated lung cancer risk among heavy smokers. This pattern is similar to our observation of a smaller lung cancer risk associated with PM for current smokers. Silverman et al. (2012) proposed a number of biological mechanisms to explain this effect, such as polycyclic aromatic hydrocarbons—present in tobacco smoke, diesel exhaust, and PM_2.5_—competing for metabolic activation, and decreased lung deposition of diesel exhaust among smokers. However, the exact mechanism leading to this reduced risk among smokers is still unclear. Thus, more careful consideration of potential interaction between PM and smoking for lung cancer and other diseases seems warranted; large, robust data sets will be needed for this work.

The original risk estimates included in our analyses assume a log-linear relation between PM exposure and lung cancer rates. Thus, the data available for this meta-analysis do not provide the opportunity to further evaluate this assumption. However, alternatives to a linear exposure–response model have been considered in analyses of data from the ACS-CPS II (Pope et al. 2002; Turner et al. 2011), Harvard Six Cities (Lepeule et al. 2012), Canadian National Enhanced Cancer Surveillance System (Hystad et al. 2013), Rome Longitudinal (Cesaroni et al. 2013), NHS (Puett et al. 2014), and ESCAPE (Raaschou-Nielsen et al. 2013) studies, which included categorical modeling and application of smoothing functions. All of these analyses concluded that there is no evidence of marked deviation from linearity. Pope et al. (2011) also reported a near linear relation of lung cancer risk with estimated daily PM_2.5_ dose in an analysis that integrated information on lung cancer risk associated findings of risk from diverse combustion sources.

## Conclusion

The results of these analyses, and the decision of the IARC Working Group to classify outdoor air pollution as a Group 1 carcinogen, further justify efforts to reduce exposures to air pollutants, which can arise from many sources. The Global Burden of Disease collaboration estimated that approximately 3.22 million deaths were caused by exposure to air pollution in 2010, an increase from 2.91 million deaths attributed to air pollution in 1990 (Lim et al. 2012). Cancers of the trachea, bronchus, or lung represent approximately 7% of total mortality attributable to PM_2.5_ in 2010. The results of the meta-analysis provided here could be useful for better quantifying the burden of lung cancer associated with air pollution. The Group I classification raises questions regarding individual components in the air pollution mixture regarding, for example, the carcinogenic potential of each component as well as through what pathways they may contribute to cancer risk.

## Supplemental Material

(184 KB) PDF
