# Erratum: “Outdoor Particulate Matter Exposure and Lung Cancer: A Systematic Review and Meta-Analysis”

**DOI:** 10.1289/ehp.122-A236

**Published:** 2014-09-01

In the original online version of “Outdoor Particulate Matter Exposure and Lung Cancer: A Systematic Review and Meta-Analysis” by Hamra et al. [Environ Health Perspect 122:906–911 (2014); http://dx.doi.org/10.1289/ehp.1408092], there were several errors. In Table 1, the number of events for Krewski et al. (2009; United States) and for Beelen et al. (2008; Netherlands) should have been “9,788 (mortality)” and “1,940 (incidence),” respectively. In Figure 1A, all of the weights were incorrect, as was the RR (95% CI) for the North America subtotal [it should have been “1.11 (1.05, 1.16)” instead of “1.10 (1.06, 1.14)”]. Finally, in Table 2, the study ID numbers for the PM_2.5_ subanalyses for North America should not have included “5”; the correct study numbers are 2, 4, 6, 7, 8, 9, and 10. All of these errors have been corrected.

The authors regret the errors.

