# Erratum: “Outdoor Particulate Matter Exposure and Lung Cancer: A Systematic Review and Meta-Analysis”

**DOI:** 10.1289/ehp.122-A294

**Published:** 2014-11-01

Hamra et al. reported an error in their article [Outdoor Particulate Matter Exposure and Lung Cancer: A Systematic Review and Meta-Analysis. Environ Health Perspect 122:906–911 (2014); http://dx.doi.org/10.1289/ehp.1408092]. In the “Results” section of the Abstract, the 95% confidence interval (CI) for the risk estimate for PM_2.5_ among former smokers was incorrect. The corrected sentence is as follows:

Analyses by smoking status showed that lung cancer risk associated with PM_2.5_ was greatest for former smokers [1.44 (95% CI: 1.04, 2.01)], followed by never-smokers [1.18 (95% CI: 1.00, 1.39)], and then current smokers [1.06 (95% CI: 0.97, 1.15)].

The authors regret the error.

