# Supplementary material for: Outdoor Particulate Matter Exposure and Lung Cancer: A Systematic Review and Meta-Analysis
Source: Environ Health Perspect. 2014 Jun 6;122(9):906–11. doi: 10.1289/ehp/1408092 (PMC4154221; doi:10.1289/ehp/1408092)
Supplement: (184 KB) PDF [file ehp.1408092.s001.pdf]

Environmental Health Perspectives doi:10.1289/ehp.1408092

Supplemental Materials

Outdoor Particulate Matter Exposure and Lung Cancer: A Systematic Review and Meta-Analysis

Ghassan B. Hamra, Neela Guha, Aaron Cohen, Francine Laden, Ole Raaschou-Nielsen, Jonathan M. Samet, Paolo Vineis, Francesco Forastiere, Paulo Saldiva, Takashi Yorifuji, and Dana Loomis

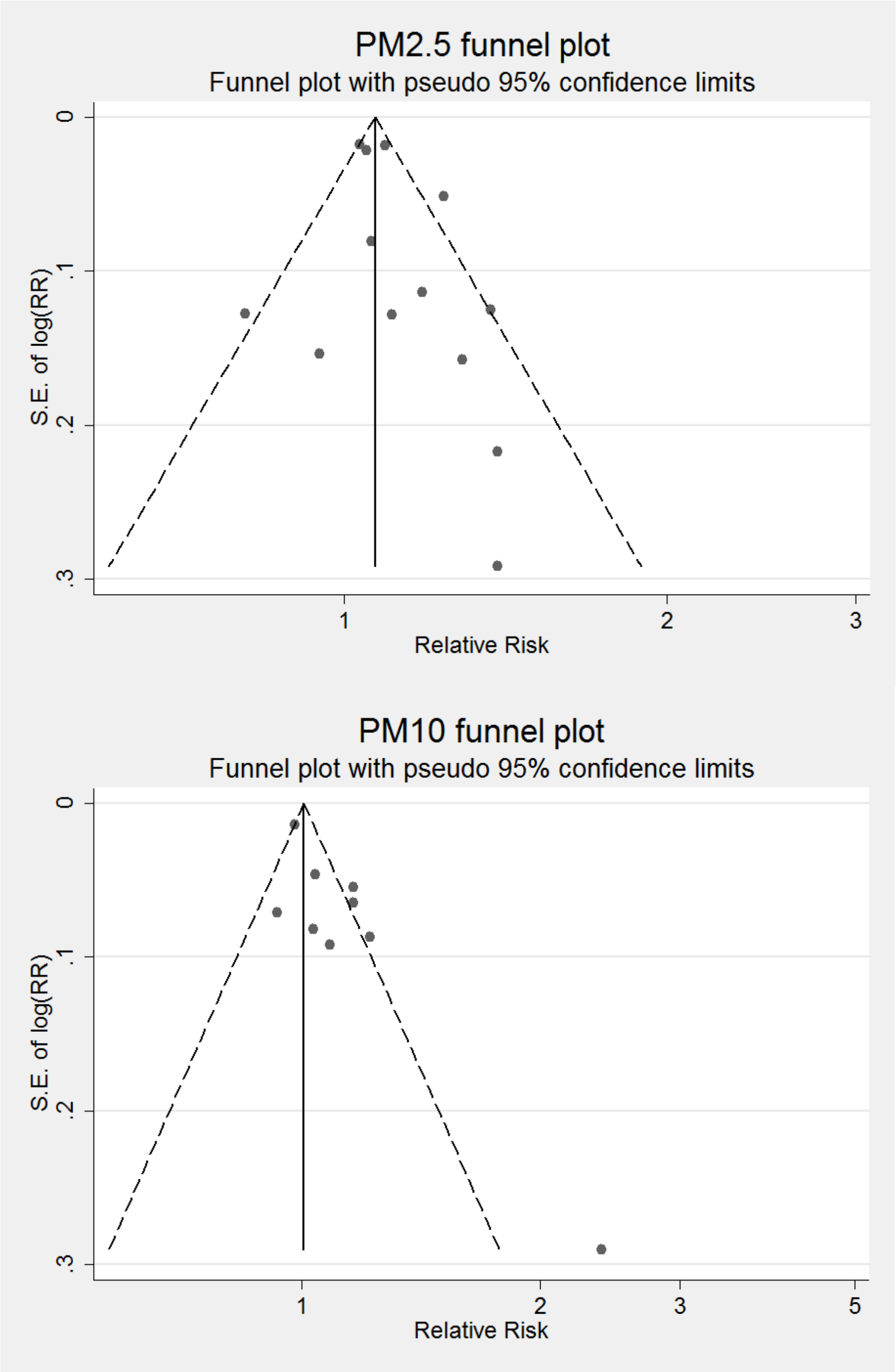

Figure S1. Funnel plots for studies of lung cancer incidence and mortality associated with $PM_{2.5}$ (top) and PM_{10} (bottom). *Center lines of the funnel plots represent a fixed effect, and not a random effect, meta-estimate; thus, they do not correspond to the estimates presented in Table 2 of the manuscript. Additionally, Jerrett et al. (2013) is excluded from the funnel plot for $PM_{2.5}$ exposure. Funnel plots were generated with STATA package metafunnel.

Table S1. Influence analyses examining the lung cancer incidence and mortality associated with a 10 μg/m^3 change in exposure to $PM_{2.5}$ and PM_{10}.

Study excluded	Location	RR (95% CI)
**$PM_{2.5}$**		
None	--	1.09 (1.04, 1.14)
Beelen et al., 2008	Netherlands	1.09 (1.05, 1.17)
Cao et al. 2011	China	1.11 (1.05, 1.17)
Carey et al. 2013	UK	1.09 (1.04, 1.14)
Cesaroni et al. 2013	Italy	1.11 (1.05, 1.17)
Hart et al. 2011	USA	1.09 (1.04, 1.14)
Hystad et al. 2013	Canada	1.09 (1.04, 1.14)
Jerrett et al. 2013	USA	1.09 (1.04, 1.14)
Katanoda et al. 2011	Japan	1.07 (1.03, 1.11)
Krewski et al. 2009	USA	1.10 (1.04, 1.16)
Lepeule et al. 2012	USA	1.08 (1.04, 1.13)
Lipsett et al. 2011	USA	1.10 (1.05, 1.15)
McDonell et al. 2000	USA	1.09 (1.04, 1.14)
Puett et al. 2013	USA	1.09 (1.04, 1.14)
Raaschou-Neilsen et al 2013	Europe	1.10 (1.04, 1.15)
**PM_{10}**		
None	--	1.08 (1.00, 1.17)
Beeson et al. 1998	USA	1.07 (0.98, 1.16)
Carey et al. 2013	UK	1.09 (1.00, 1.18)
Hales et al. 2012	New Zealand	1.07 (0.98, 1.15)
Hart et al. 2011	USA	1.08 (0.99, 1.17)
Heinreich et al. 2013	Germany	1.06 (0.99, 1.14)
Lipsett et al. 2011	USA	1.10 (1.01, 1.20)
Pope et al. 2002	USA	1.10 (1.01, 1.20)
Puett et al. 2013	USA	1.06 (0.98, 1.15)
Raaschou-Neilsen et al. 2013	Europe	1.09 (0.99, 1.20)
